# Thioredoxin reductase from *Bacillus cereus* exhibits distinct reduction and NADPH‐binding properties

**DOI:** 10.1002/2211-5463.13289

**Published:** 2021-09-23

**Authors:** Marita Shoor, Ingvild Gudim, Hans‐Petter Hersleth, Marta Hammerstad

**Affiliations:** ^1^ Department of Biosciences Section for Biochemistry and Molecular Biology University of Oslo Norway; ^2^ Department of Chemistry Section for Chemical Life Sciences University of Oslo Norway

**Keywords:** crystal structure, flavodoxin reductase, ribonucleotide reductase, thioredoxin reductase

## Abstract

Low‐molecular‐weight (low *M*_r_) thioredoxin reductases (TrxRs) are homodimeric NADPH‐dependent dithiol flavoenzymes that reduce thioredoxins (Trxs) or Trx‐like proteins involved in the activation networks of enzymes, such as the bacterial class Ib ribonucleotide reductase (RNR). During the last few decades, TrxR‐like ferredoxin/flavodoxin NADP^+^ oxidoreductases (FNRs) have been discovered and characterized in several types of bacteria, including those not encoding the canonical plant‐type FNR. In *Bacillus cereus*, a TrxR‐like FNR has been shown to reduce the flavodoxin‐like protein NrdI in the activation of class Ib RNR. However, some species only encode TrxR and lack the homologous TrxR‐like FNR. Due to the structural similarity between TrxRs and TrxR‐like FNRs, as well as variations in their occurrence in different microorganisms, we hypothesized that low *M*_r_ TrxR may be able to replace TrxR‐like FNR in, for example, the reduction of NrdI. In this study, characterization of TrxR from *B. cereus* has revealed a weak FNR activity toward NrdI reduction. Additionally, the crystal structure shows that only one out of two binding sites of the *B. cereus* TrxR homodimer is occupied with NADPH, indicating a possible asymmetric co‐substrate binding in TrxR.

## Graphical abstract

*Bacillus cereus* thioredoxin reductase (TrxR) reveals weak activity as a reductase of the flavodoxin‐like protein NrdI, as compared to the endogenous NrdI‐reductase ferredoxin/flavodoxin NADP^+^ oxidoreductase (FNR). A TrxR mutant, designed to resemble FNR, did not improve the catalytic efficiency. The crystal structure of homodimeric *B. cereus* TrxR shows a single bound NADPH, indicating a possible asymmetric co‐substrate binding in TrxR.

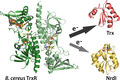

**Abbreviations**
*Bc*
: *Bacillus cereus*
Bdr: bacillithiol disulfide reductase
*Dr*
: *Deinococcus radiodurans*
*Ec*
: *Escherichia coli*
FAD: flavin adenine dinucleotide
Fld: flavodoxin
FMN: flavin mononucleotide
FNR: ferredoxin/flavodoxin NADP^+^ oxidoreductases
FO: flavin‐oxidizing conformation
FR: flavin‐reducing conformation
GR: glutathione reductase
*Hp*
: *Helicobacter pylori*
IruO: iron‐uptake oxidoreductase
*Ll*
: *Lactobacillus* *lactis*
low *M*_r_
: low‐molecular‐weight
NADPH: nicotinamide adenine dinucleotide phosphate
PDBid: Protein Data Bank identification code
RNR: ribonucleotide reductase
*Sa*
: *Staphylococcus aureus*
Trx: thioredoxin
TrxR: thioredoxin reductase

Thioredoxin reductases (TrxRs) are members of the pyridine nucleotide‐disulfide oxidoreductase family of flavoenzymes [1, 2]. As part of the thioredoxin (Trx) system, comprising nicotinamide adenine dinucleotide phosphate (NADPH), TrxR, and Trx, TrxR is a member of a major disulfide reductase system which can provide electrons to a large range of enzymes. The TrxR‐Trx system participates in multiple important biological processes. It plays a preventive role in oxidative stress in bacteria, archaea, and eukarya, and in regulation of DNA synthesis, gene transcription, cell growth, and apoptosis in bacteria, yeast, and mammals [3, 4, 5, 6]. TrxR contains a redox‐active disulfide/dithiol adjacent to a flavin adenine dinucleotide (FAD) cofactor [7] and reduces its substrate Trx through this highly conserved cysteine pair (CXXC). The regeneration is aided by the reduction of the enzyme‐bound FAD group by NADPH [8, 9].

Low‐molecular‐weight (low *M*_r_) TrxRs (˜ 35 kDa), present in bacteria, archaea, fungi, and plants [8, 10, 11, 12], are homodimeric enzymes. Each monomer consists of two globular domains, one FAD‐binding domain and one NADPH‐binding domain, composed of three‐layer ββα sandwich Rossmann‐like folds connected through a two‐stranded β‐sheet hinge region [13, 14]. The NADPH‐binding domain also harbors the redox‐active disulfide/dithiol, which is located in the conserved active‐site CXXC motif [14, 15].

In low *M*_r_ TrxRs, both the NADPH‐binding site and the CXXC motif are positioned on the *re*‐face of FAD and hence cannot simultaneously be positioned close to the FAD isoalloxazine ring. Therefore, a ˜ 67° rotation of the NADPH‐binding domain relative to the FAD‐binding domain is essential for sequential and efficient electron transfer from NADPH to FAD and from FADH_2_ to the redox‐active disulfide. Hence, the NADPH‐ and FAD‐binding domains are found in two conformational states: one where the FAD cofactor is reduced by NADPH (FR, flavin‐reducing conformation) and one where the FAD cofactor is reoxidized by the active‐site disulfide (FO, flavin‐oxidizing conformation), interchanged by the ˜ 67° rotation [14, 15, 16]. The rotation between the two domains also allows the redox‐active cysteines to interact with its substrate, Trx, and the dithiol form of Trx can further reduce its target substrate. Trx was originally discovered to be a reducing substrate of ribonucleotide reductase (RNR) [17]. This essential enzyme catalyzes the *de novo* synthesis of 2′‐deoxyribonucleotides from their corresponding ribonucleotides and is therefore crucial for DNA repair and replication in all DNA‐based living organisms [4, 5, 18, 19]. The reducing equivalents provided by the TrxR‐Trx system are essential for the catalytic activity of oxygen‐dependent class I and II RNRs.

In addition to the thiol‐based redox pathway, the bacterial class Ib RNR has been shown to rely on another redox partner, the small flavodoxin (Fld)‐like protein NrdI [20, 21, 22], needed for the formation of the Mn^III^_2_‐tyrosyl radical (Y^•^) cofactor [20]. NrdI is in turn activated by a ferredoxin/flavodoxin NADP^+^ oxidoreductases (FNR), as shown in our previous studies [23, 24] (Fig. 1). FNRs can be separated into two main families, plant‐type FNRs and glutathione reductase (GR)‐like FNRs, which are structurally and phylogenetically unrelated [25].

**Fig. 1:**
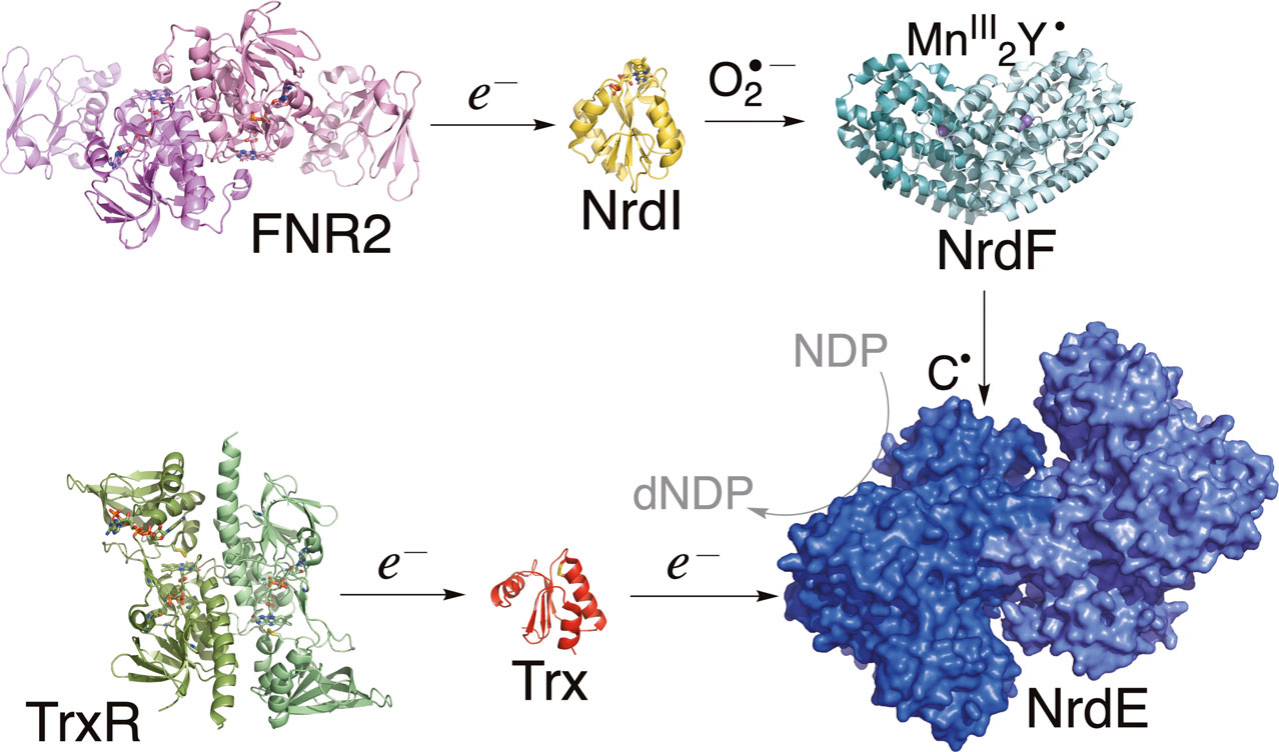
Ribonucleotide reductase class Ib activation pathway in *B. cereus*. (PDBid: 6GAS, FNR2; 2X2O, NrdI; 4BMU, NrdF; 3ZIJ, Trx‐like protein BC3987; 7AAW, TrxR).

The FNR that activates NrdI in *Bacillus cereus* (*Bc*) is a TrxR‐like FNR (a subfamily of the GR‐like FNRs). These FNRs are structurally homologous to the homodimeric low *M*_r_ TrxR with an identical organization of the NADPH‐ and FAD‐binding domains, however, showing variations in the orientation of the NADPH‐binding domain relative to the FAD‐binding domain and lacking the essential CXXC catalytic motif of TrxRs. The TrxR‐like FNRs constitute a large and diverse group of oxidoreductases, many of which have been discovered and characterized recently. These enzymes have evolved to perform a diverse set of biochemical reactions on different substrates [26]. The firmicute *B*. *cereus* encodes NrdI, two Flds, one TrxR, and three TrxR‐like FNRs, FNR1−3. To gain insight into the specificity and selectivity of the FNR‐Fld interaction, we have previously compared the interactions of the different FNRs and their potential NrdI/Fld redox partners from *B*. *cereus* [23, 24]. Our studies revealed significant differences in the catalytic efficiencies and turnover rates of the different FNR‐Fld/NrdI pairs [23, 24, 27, 28] with FNR2 showing the highest turnover number. FNR2 was therefore established as an endogenous redox partner in the activation of NrdI in class Ib RNR from *B*. *cereus*. In contrast, FNR3 has recently been shown to function as a bacillithiol disulfide reductase (Bdr) [28, 29, 30] and a *B*. *cereus* FNR1 homolog from *Staphylococcus aureus* (*Sa*) has been shown to function as an iron‐uptake oxidoreductase (IruO) [31].

The TrxR‐like FNRs are widely found in firmicutes, but can also be found in other bacterial phyla encoding the class Ib RNR and NrdI. However, for example, *Escherichia coli* (*Ec*) does not contain any TrxR‐like FNRs, *Deinococcus radiodurans* (*Dr*) only contains a Bdr and no FNR1 or FNR2 homologs, whereas the firmicute *S*. *aureus* does not contain a *B*. *cereus* FNR2 homolog. Due to the lack of TrxR‐like FNRs (in particular homologs of *B*. *cereus* FNR2) in some bacteria encoding class Ib RNR, and due to the structural similarities between FNRs and TrxR, it should be explored whether TrxR could possibly serve as an alternative redox partner to NrdI and hence activation of the class Ib RNRs. Furthermore, to understand the differences between TrxR and TrxR‐like FNRs in terms of redox partner selectivity, it is of interest to investigate which structural features could account for variations in substrate specificity.

In this work, we tried to answer whether TrxR could potentially replace TrxR‐like FNRs by monitoring their activity as reductants of NrdI. Also, by introducing mutations to TrxR, we aimed to map which structural variations in these two enzymes could explain the differences in activity toward the NrdI and Trx substrates for FNR and TrxR, respectively, and if the TrxR mutant, designed to resemble an FNR, would reveal an increased reduction rate toward NrdI as a redox partner. By choosing *B*. *cereus* as a model organism, encoding both TrxR and three TrxR‐like FNRs, a direct comparison of the reduction rates between these different enzymes could be made. By solving the crystal structure of *B*. *cereus* TrxR, we have also observed structural features indicating a possible half‐of‐the‐sites reactivity with respect to NADPH in *B*. *cereus* TrxR.

## Methods

### Cloning, protein expression, and purification

TrxR and TrxR mutant (TrxR_wt_ and TrxR_mutant_) were expressed and purified as described previously for the *B*. *cereus* FNRs [24], with some modifications. In brief, genes for TrxR_wt_ and TrxR_mutant_ (locus tag BC5159, *B*. *cereus* ATCC 14579, GenScript) were synthesized and cloned into the pET‐22b(+) plasmid using restriction enzymes NdeI and HindIII (GenScript), and transformed into competent *E. coli* BL21 (DE3) cells (Novagen). For the TrxR_mutant_, mutations were introduced by GenScript according to the description in the ‘Results and Discussion’ section. Cells were grown in Terrific Broth (TB) medium containing 100 μg·mL^−1^ ampicillin. Protein expression was induced by adding isopropyl β‐D‐1‐thiogalactopyranoside (IPTG, Thermo Scientific, Oslo, Norway) to a final concentration of 0.5 mm, and the cultures were incubated for 12–15 h at 20 °C with vigorous shaking before harvesting. The frozen cell paste was lysed either using an X‐press [32] or by sonication (1 : 5 ratio of cell wet weight‐to‐buffer volume), dissolved in 100 mm Tris/HCl, pH 7.5, 2 mm DTT, 5 μg·mL^−1^ DNase, protein inhibitor cocktail tablet (Roche, Oslo, Norway), and cleared from nucleic acids by streptomycin sulfate (2.5%). Proteins were precipitated with 0.45–0.6 g·mL^−1^ ammonium sulfate, dissolved in 50 mm Tris/HCl, pH 7.5, and desalted using a HiTrap Desalting column (GE Healthcare, Oslo, Norway). Desalted protein was applied on a HiTrap HP Q column (GE Healthcare), eluted with a 0.1–0.4 M KCl gradient, desalted as described previously, and applied on a Source 15Q column (GE Healthcare), eluted with a 0–0.3 M KCl gradient. Proteins were concentrated, aliquoted, flash‐frozen in liquid N_2_, and stored at −80 °C. Protein concentration was determined using a molar extinction coefficient of 11.3 mm ^−1^·cm^−1^ for the protein‐bound flavin at 450 nm [33].

### Protein crystallization

All initial crystallization screening was performed with a Mosquito crystallization robot (TTP Labtech). TrxR_wt_ crystals (12 mg·mL^−1^) were obtained using the sitting drop vapor diffusion method at 4 °C with 14% (w/v) PEG 5000 MME, 0.1 M magnesium acetate, and 0.1 M sodium citrate, pH 5.8 (MemGold Screen, Molecular Dimensions, Ltd., Sheffield, UK). Crystals were briefly soaked in cryoprotectant solution (30% glucose, 14% (w/v) PEG 5000 MME, 0.1 M magnesium acetate, and 0.1 M sodium citrate, pH 5.8) and flash‐cooled in liquid N_2_.

### Crystal data collection and refinement

Diffraction data were collected at beamline ID23‐1 at the European Synchrotron Radiation Facility (ESRF), Grenoble, France. The dataset was processed with MOSFLM [34], scaled, and merged using AIMLESS [35, 36], and the space group confirmed with POINTLESS through the CCP4 software suite [37]. The TrxR_wt_ structure was solved by molecular replacement using PHASER [38]. A homolog model of *B*. *cereus* TrxR_wt_ modeled with SWISS‐MODEL [39, 40] from the *S*. *aureus* TrxR Protein Data Bank identification code (PDBid: 4GCM) was used as a search model in PHASER to solve the *B*. *cereus* TrxR_wt_ structure. Initial refinement was done using restrained refinement in REFMAC5 [41] followed by several cycles of refinement with phenix.refine [42] in the Phenix suite [43] and model building in Coot [44]. All structure figures were prepared using PyMOL (Schrödinger, LLC, New York, NY, USA).

The standard Phenix restraints used for the FAD cofactor were modified to take into account potential X‐ray radiation‐induced reduction of the FAD cofactor making the isoalloxazine ring free to bend along the N5–N10 axis (butterfly bend) [27]. Similarly, the disulfide bond restrains were loosened to take into account potential reduction. The angle of the butterfly bend of the isoalloxazine ring was calculated with the psico module in PyMOL, by calculating the angle between the two planes defined by atoms N5, C4X, C4, N3, C2, C10, N10, and N5, C5X, C3, C7, C8, C9, C9A, and N10. A slight butterfly bend of 3.1° and 4.8° was observed for the isoalloxazine rings, consistent with expected radiation‐induced reduction. The lengths of the disulfide bonds were refined to 2.5 and 2.3 Å (for monomer 2 and 1, respectively), indicating only a slight elongation from a disulfide bond of 2.04 Å due to radiation‐induced reduction [45, 46]. The largely intact disulfide bond can also be seen from the clear electron density between the sulfur atoms with no significant electron difference density. The absorbed X‐ray dose was calculated with the program RADDOSE‐3D [47].

### Steady‐state kinetic measurements

To measure the ability of TrxR_wt_ and the TrxR_mutant_ to reduce NrdI, steady‐state kinetic measurements were performed. The experimental procedure and data analysis were performed essentially as described previously [23, 24]. In brief, the reactions were carried out with 200 μm NADPH, 0.5 μm TrxR_wt_ or TrxR_mutant_, and various concentrations of NrdI (2.5–20 μm). NADPH and TrxR were incubated and stirred for 15 min, and NrdI was added to start the reaction. The reduction of NrdI was monitored by the disappearance of the NrdI_ox_ state at *λ*_max_ = 447 nm and the appearance of the NrdI_sq_ state at *λ*_max_ = 575 nm. Due to NrdI’s high reactivity with dioxygen, the steady‐state experiments were carried out in a glove box (Plas Labs 855‐AC) under strict anaerobic conditions (91% N_2_, 9% H_2_, AGA) using an Agilent 8453 UV‐visible spectrophotometer.

### Bioinformatic analysis

Selected bacterial TrxR and TrxR‐like FNR protein sequences were obtained from the National Center for Biotechnology Information resources and used for multiple sequence alignments. The selected bacteria were from firmicutes containing both TrxR and TrxR‐like FNRs: *Bc*, *Bacillus subtilis* (*Bs*), *Sa*, and *Lactococcus lactis* (*Ll*), in addition to TrxR from *Ec*, *Helicobacter pylori* (*Hp*), and *Dr*. The two latter organisms have TrxR structures in the PDB that contain either two or no NADPH molecules bound per homodimer, respectively. Multiple sequence alignments were performed with Clustal Omega and phylogenetic tree analysis with average distances using the BLOSUM62 matrix. The figures were generated in JalView, and the sequence alignments were colored by % identity [48]. The locus tags for the sequences used are shown in parentheses for *Sa* FNR1 (SACOL2369), *Bc* FNR1 (BC0385), *Bc* FNR2 (BC4926), *Bs* FNR/YumC (BSU32110), *Bs* FNR/YcgT (BSU03270), *Ll* TrxR (llmg_0776), *Sa* TrxR (SACOL0829), *Bc* TrxR (BC5159), *Bs* TrxR (BSU34790), *Ll* TrxR (llmg_1588) *Ec* TrxR (b0888), *Hp* TrxR (HP_0825), and *Dr* TrxR (DR_1982).

## Results and Discussion

### TrxR and FNR differences with respect to sequence and structure

**Fig. 2:**
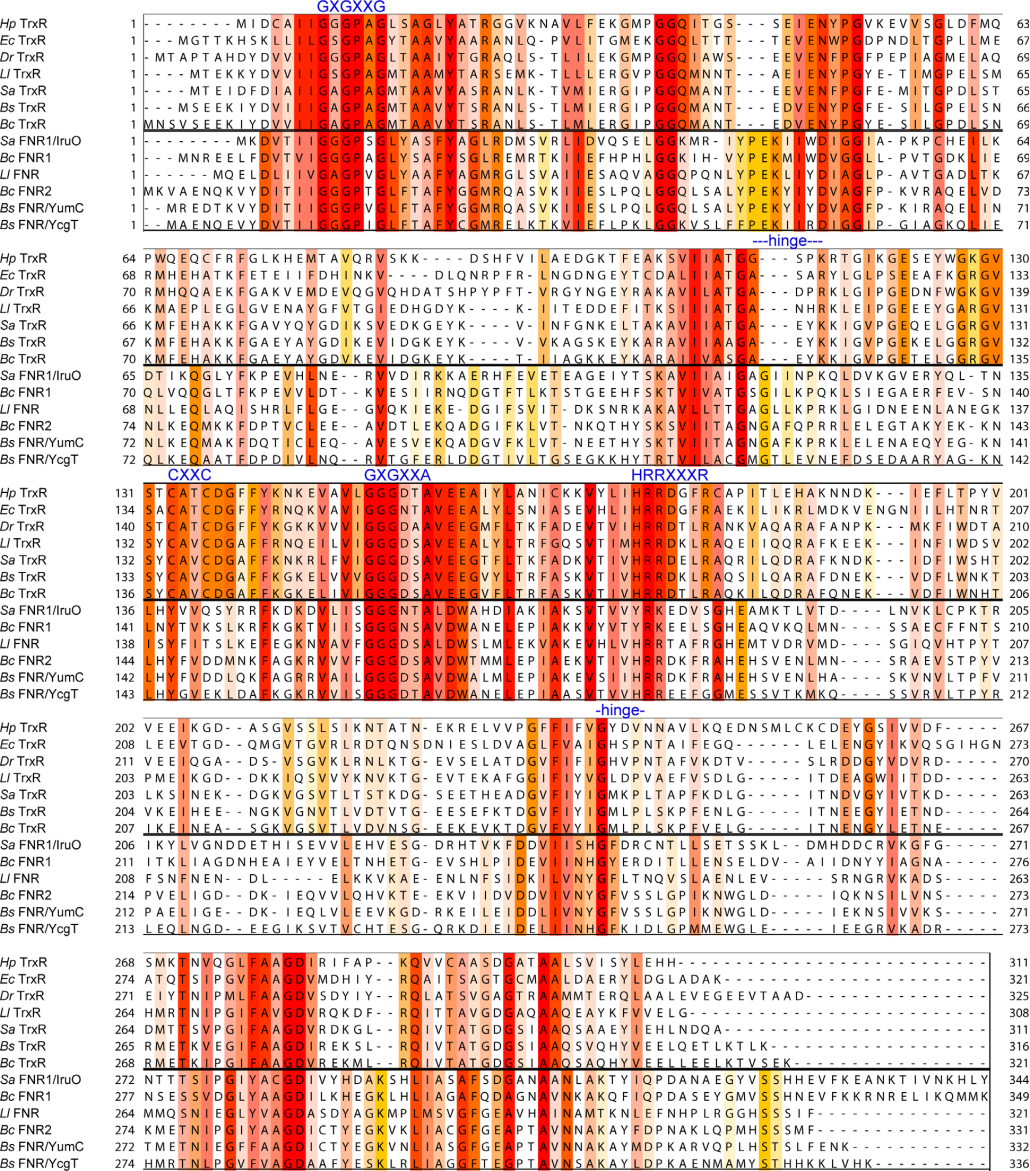
Multiple sequence alignment of selected bacterial TrxRs and FNRs from *B. cereus*, *B. subtilis*, *S. aureus*, *L*. *lactis*, *D*. *radiodurans*, *H. pylori,* and *E. coli*. Multiple sequence alignment generated with Clustal Omega through Jalview. The coloring is according to % identity. The sequences are grouped according to the phylogenetic tree in Fig. 3. Characteristic TrxR and FNR motifs are highlighted in blue above the sequence alignments.

**Fig. 3:**
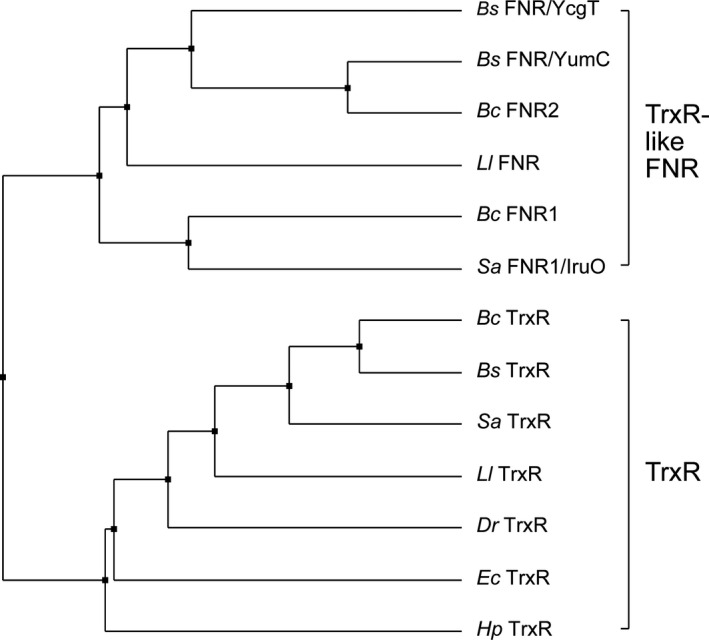
Phylogenetic tree analysis of selected bacterial FNRs and TrxRs from *B. cereus*, *B. subtilis*, *S. aureus*, *L*. *lactis*, *D*. *radiodurans*, *H. pylori*, and *E. coli*. Phylogenetic tree calculated with Jalview with average distances using the BLOSUM62 matrix on the sequence alignment in Fig. 2.

In an initial attempt to understand which residues are most important in differentiating the TrxR and FNR functions as reductases of Trx and Fld/NrdI, respectively, *B*. *cereus* TrxR was mutated and designed to resemble an FNR. As noted above, TrxR undergoes a domain rotation for successive positioning of the CXXC motif and NADPH close to the FAD isoalloxazine ring, while FNR undergoes a rotation to alter between a close positioning between NADPH and FAD, versus exposing the binding site close to the FAD isoalloxazine ring for NrdI/Fld. Hence, the requirement of a rotation is different for TrxR and TrxR‐like FNRs. Therefore, the sequences of selected TrxRs and TrxR‐like FNRs were compared to investigate particular differences in the regions most likely involved in the rotation. A sequence alignment and phylogenetic tree of selected TrxRs and TrxR‐like FNRs can be seen in Figs 2 and 3. The CXXC motif and the common sequence motifs HRRXXXR for the 2′phosphate group of NADPH and GXGXXA/G and GXGXXG for the pyrophosphate groups of NAD(P)H and FAD, respectively, are also highlighted [26] Three extra residues in one of the hinge regions are conserved among FNRs and absent in TrxRs. This extended linker is hypothesized to play a role in the rotation of the NADPH‐domain in FNRs and could be important for access and reduction of NrdI/Fld. By introducing the linker in TrxR, the ability of TrxR to bypass the disulfide active site and shuffle electrons directly from FAD to the flavin mononucleotide (FMN) cofactor of the NrdI substrate can be examined. The changes introduced to the sequence of *B*. *cereus* TrxR_mutant_, also depicted in Fig. 2, include the following: (a) insertion of an extra linker between the FAD and NADPH‐binding domains named ‘GAF’, consisting of tree amino acids; glycine, alanine, and phenylalanine (conserved in FNRs including *B*. *cereus* FNR2 and *Bacillus subtilis* FNR/YumC); (b) mutation of the redox‐active cysteines to serines; and (c) mutation of residue 320 (glutamate to serine), which is shown to form a hydrogen bond with the N5 atom on FAD in FNRs. In the latter, this residue is situated on the C‐terminal loop, protruding over and stabilizing the FAD cofactor of the neighboring subunit, whereas in TrxRs, the C‐terminal helix extends to the surface of the protein in the dimer interface, distant from the FAD cofactors [26, 49, 50].

### Kinetics of NrdI reduction

**Fig. 4:**
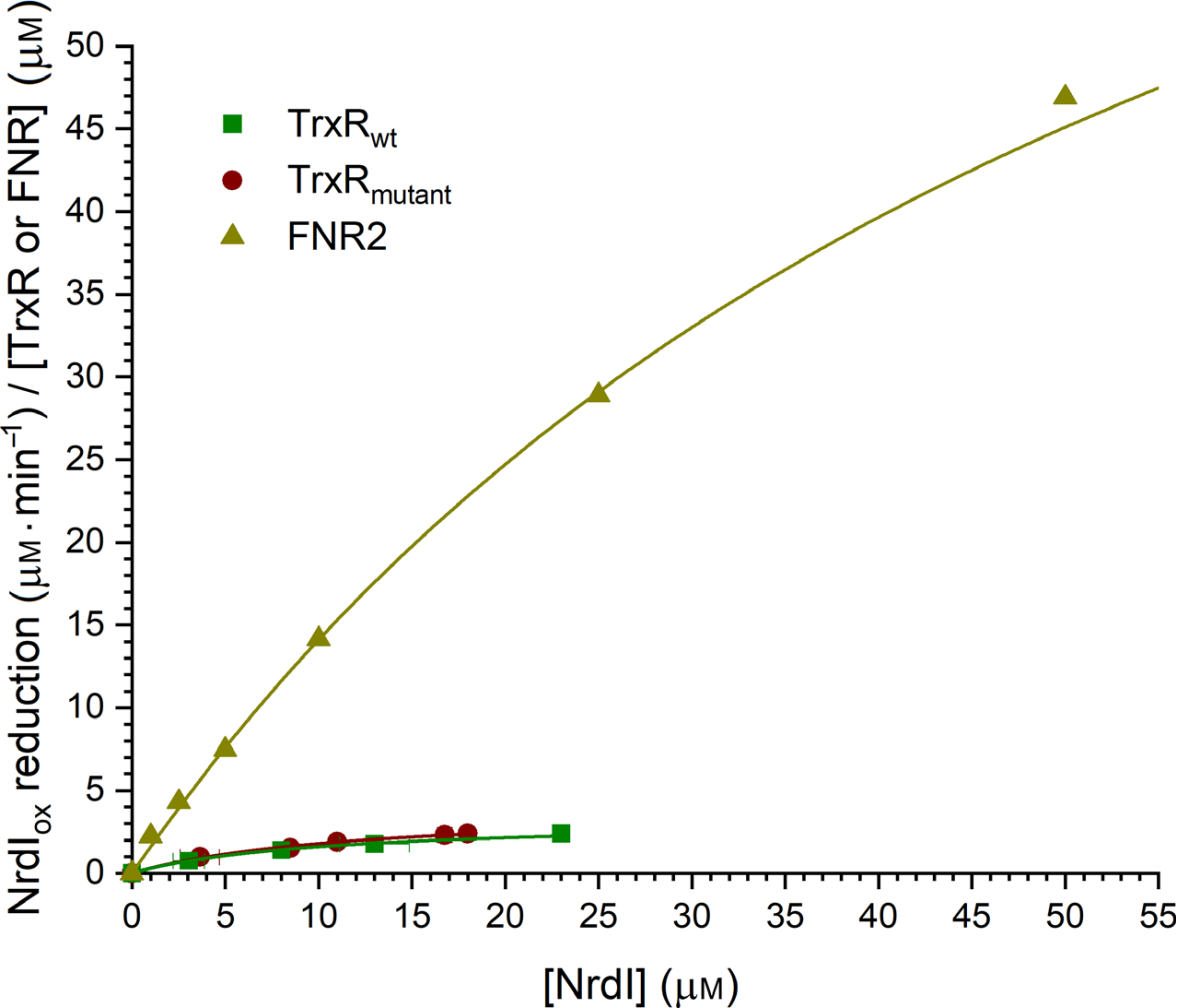
Steady‐state reduction of NrdI_ox_ by TrxR_wt_ (green, squares), and TrxR_mutant_ (dark red, circles). The reduction of NrdI_ox_ by FNR2 (olive, triangles) is included as comparison [24]. The data are fitted with the Michaelis−Menten function.

To investigate TrxR‐NrdI as an electron transfer pair, the rates of reduction of NrdI by TrxR_wt_ and TrxR_mutant_ were measured and compared to the published reduction rates of NrdI by *B*. *cereus* FNR1, FNR2, and Bdr (FNR3) [24] (Table 1 and Fig. 4). The results showed that TrxR_wt_ is able to reduce NrdI; however, the steady‐state kinetic measurements reveal that NrdI achieves a similar turnover rate with TrxR_wt_ as with the least efficient FNR in *B*. *cereus*, namely Bdr, with a *k*_cat_ of 3.2 min^−1^ versus *k*_cat_ of 2.94 min^−1^, respectively [24]. Although TrxR is able to reduce NrdI, *B*. *cereus* FNR2 has been shown to reduce NrdI at a significantly higher rate, ˜ 30‐fold higher than for the TrxR‐NrdI electron transfer pair investigated in this study (Fig. 4). Regardless, the weak turnover rate seen for the reduction of NrdI by TrxR may imply that TrxR could possibly function as a reductase of NrdI, and hence* in the activation of class Ib RNR under specific conditions or salvage modes. Further studies are needed to explore and unveil alternative and more efficient NrdI reductases in bacteria encoding the class Ib RNR, however, lacking the TrxR‐like FNR2 homolog. Moreover, the mutations introduced in the TrxR_mutant_ in this study show no significant increase in the NrdI reduction rate, with a *k*_cat_ of 3.9 min^−1^ versus *k*_cat_ of 3.2 min^−1^ for the TrxR_mutant_ and TrxR_wt_, respectively. Therefore, other aspects associated with the FNR/TrxR structures could be more crucial and should be considered in order to elucidate important features responsible for the substrate specificity in TrxRs and FNRs and for efficient FNR‐NrdI reduction.

**Table 1:** Steady‐state kinetic parameters for the reduction of NrdI_ox_ by FNR/Bdr/TrxR from *B. cereus*.

Enzyme	NrdI (substrate)		
	*k*_cat_ (min^−1^)	*K*_M_ (μm)	*k*_cat_/*K*_M_ (μm ^−1^·min^−1^)
FNR2^a^	100 ± 4	61 ± 5	1.6 ± 0.2
FNR1^a^	8.0 ± 0.1	2.7 ± 0.2	3.0 ± 0.2
Bdr^a^	2.94 ± 0.04	0.74 ± 0.06	3.9 ± 0.4
TrxR_wt_	3.2 ± 0.6	10 ± 4	0.3 ± 0.1
TrxR_mutant_	3.9 ± 1.1	12 ± 7	0.3 ± 0.1

^a^ Lofstad *et al*. 2016[24].

Although the turnover rate of NrdI reduction presented in this work is low with TrxR, previous studies have confirmed the role of *B*. *cereus* TrxR as a reductase of the Trx‐like redoxin NrdH (BC3987) from *B*. *cereus* (Fig. 1) and verified the role of these redox partners in the activation of class Ib RNR from *B*. *cereus* [51]. *Bacillus anthracis* NrdH, as well as Trx1, have also been proven to serve as redox partners for *B. anthracis* TrxR (sequence identities with *B*. *cereus* NrdH, Trx, and TrxR are 97%, 100%, and 98%, respectively) [51, 52].

### The overall structure of *B*. *cereus* TrxR and presence of NADPH

**Fig. 5:**
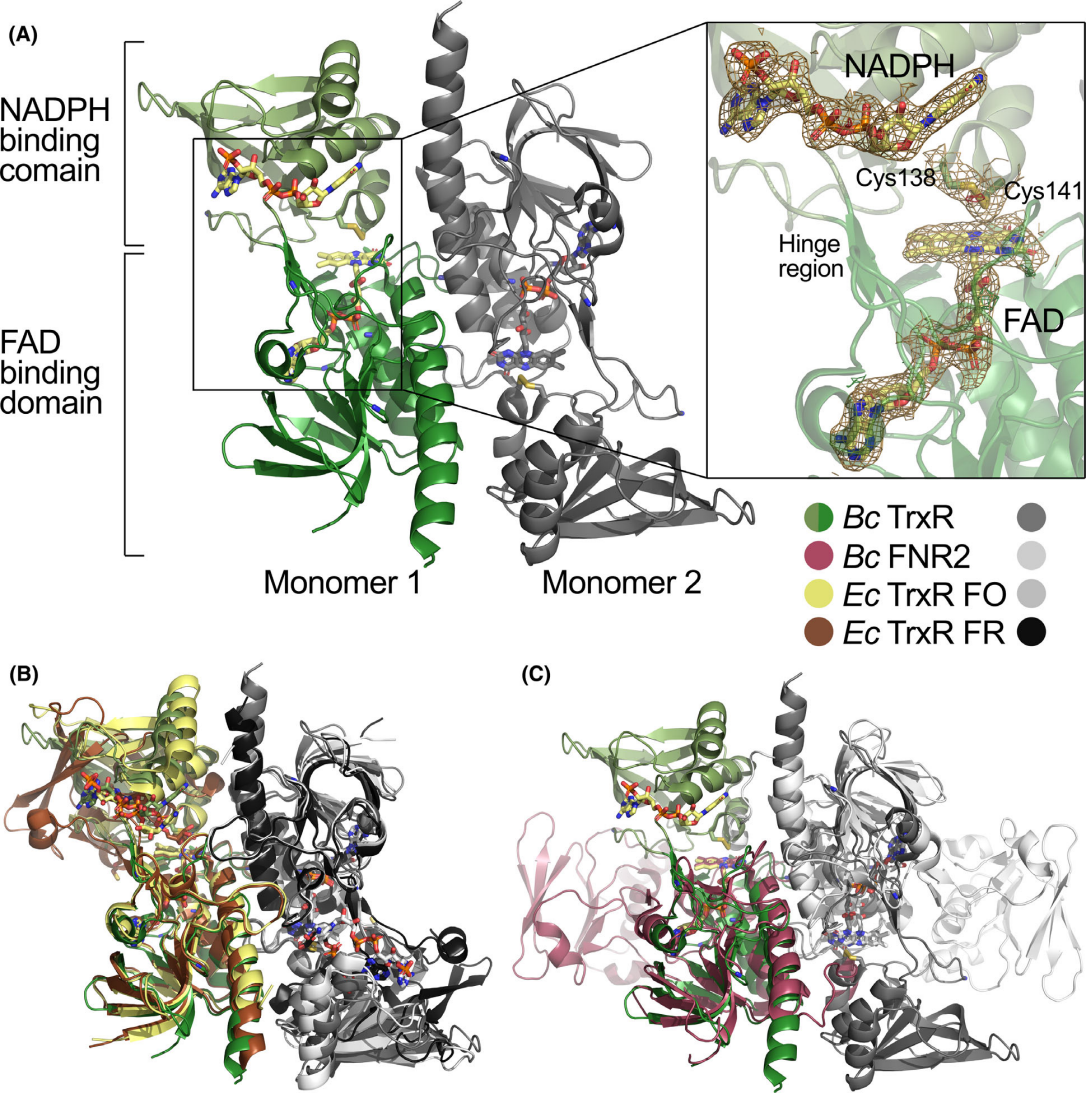
(A) Crystal structure of the low *M*_r_ TrxR homodimer from *B. cereus* (PDBid: 7AAW) with the two monomers colored in green and gray, respectively. The structure is in the FO form. The NADPH‐binding domains and FAD‐binding domains are shown in light and dark green, respectively, with the cofactors NADPH and FAD, as well as the redox‐active Cys138‐Cys141 pair shown as sticks and colored by atom type. NADPH is only bound in monomer 1. Inset: electron density maps shown around NADPH, FAD, and cystine of monomer 1. The 2F_o_–F_c_ map is contoured at 1σ (sand), and the F_o_–F_c_ difference map is contoured at ± 3σ (green/red); (B) overlay of *B. cereus* TrxR (green/dark gray) with *E. coli* TrxR FO conformation (yellow/light gray, PDBid: 1TDF) and *E. coli* TrxR FR conformation (brown/black, PDBid: 1F6M); (C) structural alignment of the FAD‐binding domains of the *B. cereus* FNR2 and TrxR homodimers (PDBid: 6GAS and 7AAW). The FNR2 and TrxR monomers are colored in burgundy and light gray, and in green and dark gray, respectively. The NADPH‐binding domains exhibit different orientations relative to the FAD‐binding domains in the two structures.

The *B*. *cereus* TrxR structure was solved and refined to a resolution of 2.25 Å with one TrxR homodimer in the asymmetric unit (Table 2). TrxR has crystallized in the FO conformation, where the disulfide active site is located on the *re*‐face of the flavin cofactor, suitable for electron transfer from FADH_2_ to the redox‐active disulfide, which will require the rotation of the NADPH‐binding domain for reduction of FAD by NADPH and reaction with the Trx substrate in the FR conformation (Fig. 5A,B). As seen in Fig. 5A (inset), a well‐defined electron density is observed for the FAD, disulfide bond, and NADPH/NADP^+^ (monomer 1). The *B*. *cereus* TrxR structure (FO) shows high structural similarity to *E. coli* low *M*_r_ TrxR (FO) and to the FAD‐binding domain dimer of the *E. coli* low *M*_r_ TrxR (FR) but with a rotation of the NADPH‐binding domain as shown in Fig. 5B [14, 15]. Compared to the structurally related *B*. *cereus* FNR2 [23] (with the highest NrdI turnover rate), a clear conformational difference in the rotation of the NADPH‐binding domains is seen in the structure, where the FNR2 has a more open conformation for NrdI/Fld binding close to the FAD (Fig. 5C).

**Table 2:** Crystal data collection and refinement statistics.

	*B. cereus* TrxR
Data collection	
X‐ray source	ID23‐1 ESRF
Detector	Pilatus 6M F
Wavelength (Å)	0.9686
Space group	P4_1_2_1_2
*a*, *b*, *c* (Å)	95.0, 95.0, 214.6
α, β, γ (°)	90, 90, 90
Type	Standard rotation
Rotation range per image (°)	0.15
Total rotation range (°)	67.5
Exposure time per image (s)	0.092
Flux (ph/s)/Transmission (%)	6.3⋅10^11^/100
Beam size (µm^2^)	45 × 30
Crystal size (µm^3^)	100 × 100 × 200
Absorbed X‐ray dose (MGy)	
Average dose (exposed regions)	0.89
Av. diffraction weighted dose	1.77
Mosaicity (°)	0.18
Resolution range (Å)	46.72–2.25 (2.32–2.25)
Total no. of reflections	126162
No. of unique reflections	47043
*R*_meas_	0.076 (1.075)
*R*_merge_	0.068 (0.870)
Completeness (%)	99.3 (100.0)
Multiplicity	5.0 (5.2)
*<I*/σ(*I*)>	14.4 (1.7)
CC_1/2_	0.999 (0.608)
Refinement statistics	
*R*_work_/*R*_free_	17.9/21.9
Mean protein/ligands/solvent isotropic *B* factor (Å^2^)	47.4/47.3/49.8
Protein assembly in asymmetric unit (AU)	2 monomers
Protein residues in gene	321
Total modeled residues in AU	
Protein residues by chain	A: 315; B: 311
Ligands	A: 1FAD; B: 1FAD, 1NADPH
Added waters	209
Matthews coefficient	3.3
Solvent content (%)	62.3
Ramachandran favored/allowed/outliers (%)	98.2/1.6/0.2
RMSD bond lengths (Å)	0.007
RMSD bond angles (°)	0.89
Estimated overall coordinate error based on maximum likelihood (Å)	0.26
PDB ID	7AAW

**Fig. 6:**
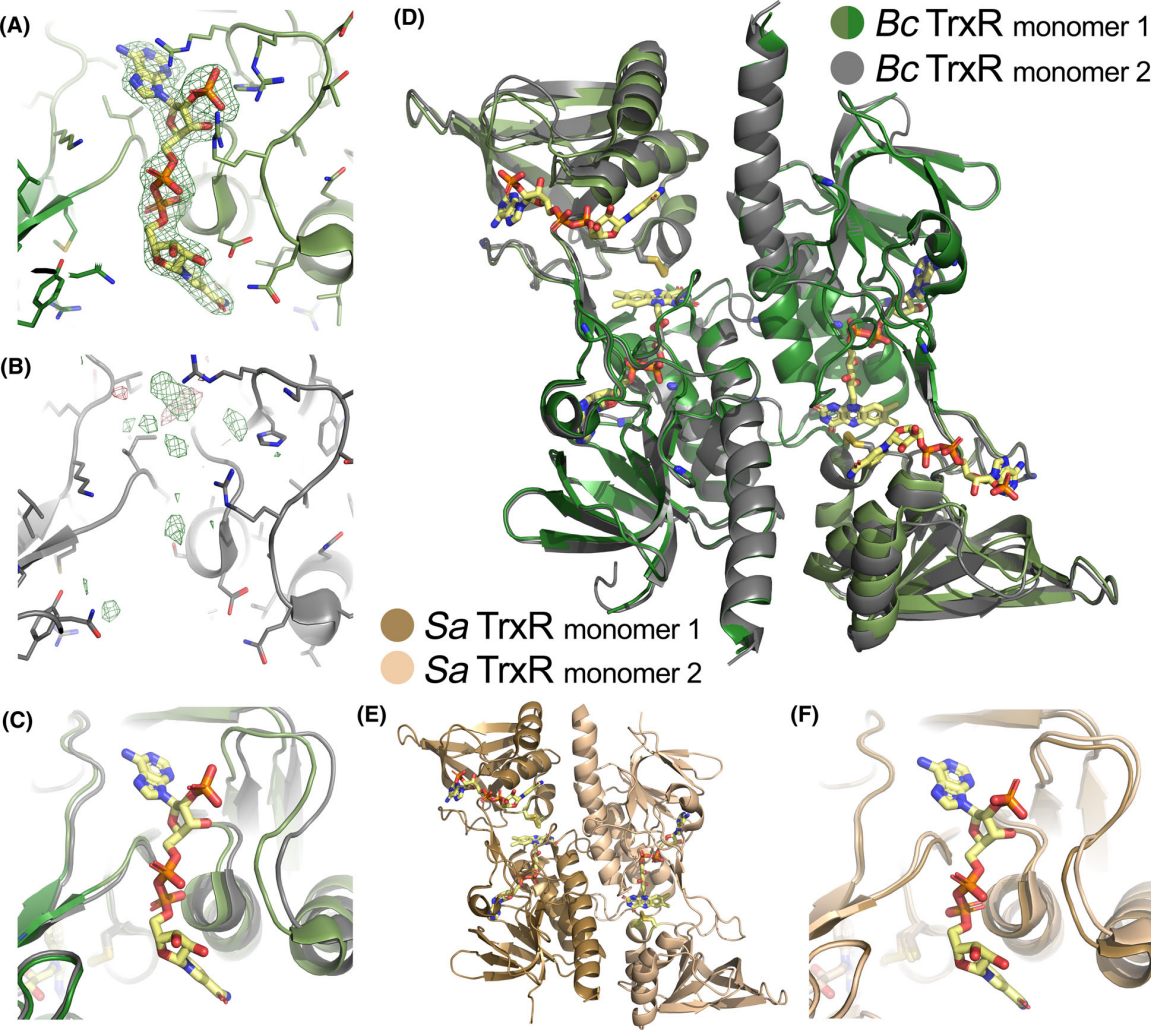
Comparison of the *B. cereus* and *S. aureus* TrxR monomer structures with respect to NADPH‐binding (PDBid: 7AAW and 4GCM). (A) *B. cereus* TrxR monomer 1 shows full electron density for NADPH, and the OMIT electron density map is contoured at + 3σ in green; (B) *B. cereus* TrxR monomer 2 shows no electron density for NADPH, the electron density difference map is contoured at ± 3σ in green/red; (C) overlay of the NADPH‐binding site of the two *B. cereus* TrxR monomers showing a change of the binding site upon NADPH‐binding; (D) overlay of the overall *B. cereus* TrxR monomers showing a movement of the NADPH‐binding domain upon binding of the cofactor; (E) structure of the *S. aureus* TrxR homodimer showing similar binding of NADPH to only monomer 1, as seen in *B. cereus* TrxR; (F) overlay of the NADPH‐binding site of the two *S. aureus* TrxR monomers showing a change of the binding site upon NADPH‐binding.

**Fig. 7:**
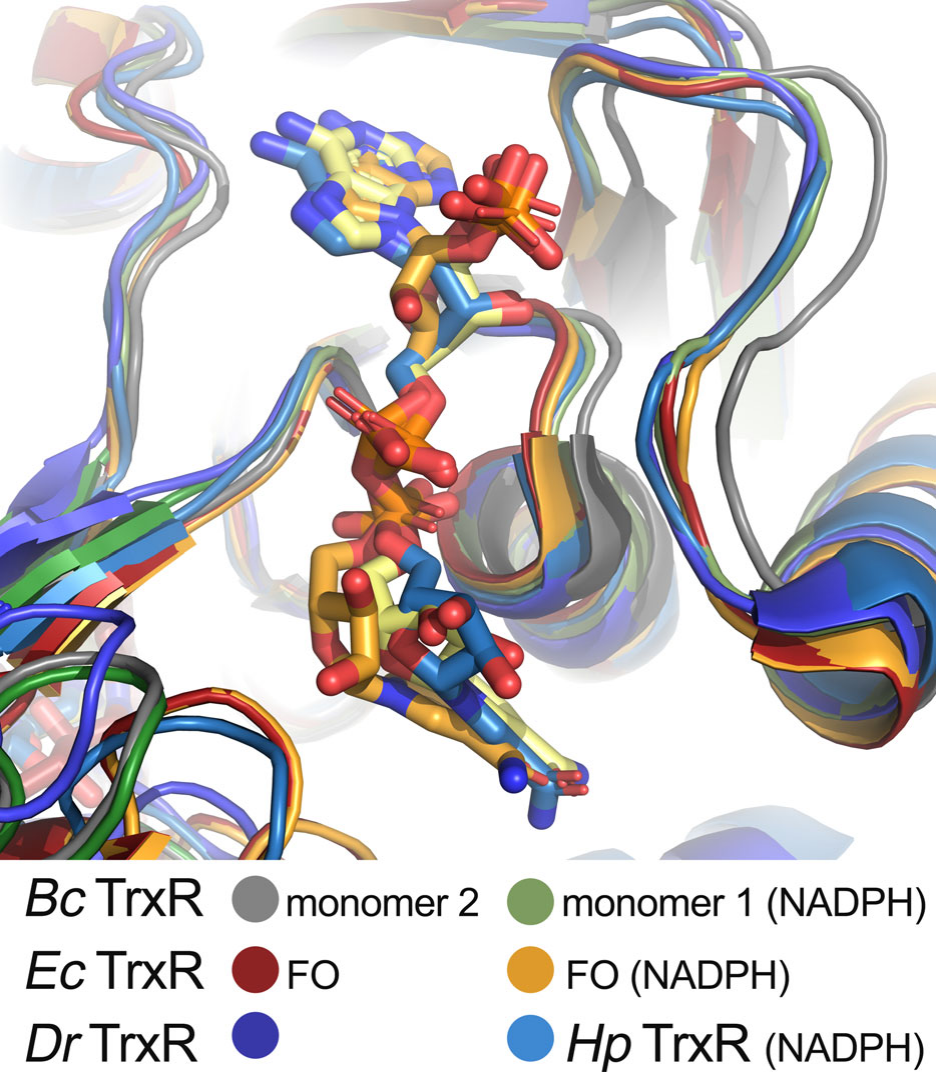
Comparison of the NADPH‐binding pocket in different TrxRs. *B. cereus* TrxR without bound NADPH in monomer 2 and bound NADPH in monomer 1 (PDBid: 7AAW), *E. coli* TrxR FO form (one monomer per asymmetric unit) without bound NADPH (PDBid: 1TDE) and with bound NADPH (PDBid: 1TDF), *H. pylori* TrxR (one dimer per asymmetric unit) without bound NADPH (PDBid: 2Q7V), *D*. *radiodurans* TrxR (one dimer per asymmetric unit) with bound NADPH in both monomers (PDBid: 2Q0K).

NADPH acts as a co‐substrate for TrxR, and some TrxR structures have been crystallized with NADPH/NADP^+^ bound, whereas others without NADPH/NADP^+^ bound. For *B*. *cereus* TrxR, no NADPH/NADP^+^ was supplemented during purification or crystallization. Unexpectedly, the structure of the TrxR dimer showed clear electron density for NADPH/NADP^+^ in monomer 1 as seen from the OMIT map in Fig. 6A, while there is no electron density for NADPH/NADP^+^ observed in the binding site in monomer 2 (Fig. 6B). A survey of the published low *M*_r_ TrxR structures in the Protein Data Bank showed that some TrxRs have crystallized with one monomer in the asymmetric unit and hence, containing either 0 or 1 NADPH/NADP^+^ molecule bound. Others have crystallized with the dimer in the asymmetric unit containing either 0 or 2 NADPH/NADP^+^ bound. The only exception is the structure of TrxR from the firmicute *S*. *aureus*, which is the TrxR most homologous to *B*. *cereus* TrxR (sequence identity 71%), which only has NADPH/NADP^+^ bound in one of the monomers (Fig. 6E,F). Overlaying the two NADPH‐binding sites in monomers 1 and 2 in *B*. *cereus* TrxR shows that the loop on one side of the NADPH‐binding site containing the HRRXXXR motif (right side in Fig. 6C) is closing in on the NADPH cofactor in monomer 1, while the loop creates a wider opening in monomer 2. This structural change on one side of the NADPH‐binding site is also seen in the overlay of the two *B*. *cereus* TrxR monomers in Fig. 6D, while no other structural changes are observed. In these two pathogenic firmicutes (*B*. *cereus* and *S*. *aureus*), only half of the sites contain NADPH/NADP^+^ fully bound, and a structural change is observed. This raises the question if the binding of the NADPH leads to the protein closing in on NADPH in an induced fit manner, or if the binding of NADPH to one site leads to a structural change so that the binding site in the other monomer opens up more and consequently increases the dissociation constant for NADPH binding to that site. A comparison to the NADPH‐binding sites in other low *M*_r_ TrxRs with and without NADPH bound shows that these structures have the same conformation of the NADPH‐binding site as *B*. *cereus* TrxR with NADPH bound, in respect to the beforementioned loop, regardless if these structures have NADPH bound or not (Fig. 7). This may point toward an opening of the loop in monomer 2 upon binding of NADPH in monomer 1, although it cannot be ruled out that the asymmetry of the two sites already exists in the NADPH‐apo form [53]. Together, these observations may possibly imply a so‐called half‐of‐the‐sites reactivity, which could be a case of extreme negative cooperativity [53, 54]. It can, however, not be ruled out that the pure half‐of‐the‐site reactivity is a crystallographically induced effect of a possible weak negative cooperativity in solution or a preexisting asymmetry, thereby not exhibiting cooperativity [53, 54]. Nevertheless, half‐site reactivity or subunit asymmetry is observed in several other pyridine nucleotide‐disulfide oxidoreductases [55]. The two conformational states of TrxR, FO and FR, are interchanged by the ˜ 67° rotation and could also lead to alternating NADPH binding and catalysis within the TrxR dimer, functioning in a sequential mode of action. These structural observations must be further confirmed by binding studies and kinetic experiments to prove whether this TrxR has an asymmetric and sequential reactivity where NADPH binds to one of the monomers at a time.

## Conclusions

We have, by using *B*. *cereus* as a model system encoding TrxR and three TrxR‐like FNRs, shown that TrxR is able to reduce NrdI, a flavoprotein crucial for the activation of class Ib RNR, however, at a very low turnover rate as compared to the suggested endogenous NrdI reductase in *B*. *cereus*. Therefore, TrxR does not seem to be a probable endogenous redox partner for NrdI, indicating that a search for more efficient redox partners for NrdI in bacteria lacking TrxR‐like FNR2 homologs should continue. A mutation of the hinge region between the NADPH‐ and FAD‐binding domains making TrxR more similar to FNR did not change the NrdI reduction capability of TrxR, showing that this change is not enough to increase the NrdI turnover rate by TrxR. In our *B*. *cereus* TrxR structure, NADPH is found in only one of the monomers of the TrxR dimer, similar to what is observed in *S*. *aureus* TrxR, however, different to what is observed in other low *M*_r_ TrxR structures. This could indicate that certain pathogenic firmicutes have adopted to an asymmetric mode of enzyme activation and successive substrate reduction by TrxR.

## Conflict of interest

The authors declare no competing financial interest.

## Author contributions

HPH and MH designed the overall experimental work, supervised the work, analyzed the data, and wrote the manuscript. MS performed the experimental work and interpreted the data. IG supervised the work and participated in data analysis. The authors have given approval to the final version of the manuscript.

## Data Availability

*B. cereus* TrxR (UniProt Accession number Q815J1). X‐ray coordinates and structure factors have been deposited in the Protein Data Bank as entry 7AAW.
